# Hydrogen peroxide route to Sn-doped titania photocatalysts

**DOI:** 10.1186/1752-153X-6-113

**Published:** 2012-10-05

**Authors:** Václav Štengl, Tomáš Matys Grygar, Jiří Henych, Martin Kormunda

**Affiliations:** 1 Department of Solid State Chemistry and Analytical Laboratory, Institute of Inorganic Chemistry AS CR v.v.i., 250 68, Řež, Czech Republic; 2 Faculty of the Environment, J.E. Purkyně University, Králova Výšina 7, 400 96, Ústí nad Labem, Czech Republic; 3 Department of Physics, Faculty of Science, J.E. Purkyně University, České mládeže 8, 400 96, Ústí nad Labem, Czech Republic

**Keywords:** TiO_2_, Sn doping, Wet synthesis, Vis light, Photocatalysis

## Abstract

**Background:**

The work aims at improving photocatalytic activity of titania under Vis light irradiation using modification by Sn ions and an original, simple synthesis method. Tin-doped titania catalysts were prepared by thermal hydrolysis of aqueous solutions of titanium peroxo-complexes in the presence of SnCl_4_ or SnCl_2_ using an original, proprietary "one pot" synthesis not employing organic solvents, metallo-organic precursors, autoclave aging nor post-synthesis calcination. The products were characterized in details by powder diffraction, XPS, UV–vis, IR, and Raman spectroscopies, electron microscopy and surface area and porosity measurements

**Results:**

The presence of tin in synthesis mixtures favors the formation of rutile and brookite at the expense of anatase, decreases the particle size of all formed titania polymorphs, and extends light absorption of titania to visible light region >400 nm by both red shift of the absorption edge and introduction of new chromophores. The photocatalytic activity of titania under UV irradiation and >400 nm light was tested by decomposition kinetics of Orange II dye in aqueous solution

**Conclusions:**

Doping by Sn improves titania photoactivity under UV light and affords considerable photoactivity under >400 nm light due to increased specific surface area and a phase heterogeneity of the Sn-doped titania powders.

## Background

In the last two decades researchers have systematically tried to improve performance of titania photocatalysts and make its synthesis simpler and more robust. Increasing titania efficiency under visible light activation has been one of the most desired improvements. There are several ways to achieve this goal, of which about the most powerful is titania modification by other elements, such as Sn. Perhaps a seminal work on this topic was published in 1995 by Vinodgopal and Kamat [1], who observed that UV-activated degradation of an azo dye by a physical mixture of SnO_2_ and TiO_2_ is by nearly an order of magnitude faster than with pure oxide components and attributed this effect to the existence of physical contacts between the two semiconductors. Similarly substantial, nearly tenfold increase in the rate of UV-activated degradation of acetone (with respect to pure rutile) was reported with Sn-doped rutile by Lin et al. [2], but they stated explicitly that the presence of segregated SnO_2_ has a detrimental effect on the catalysis. Those studies have been followed by many other researchers; they mostly revealed that the titania lattice substitution by Sn improves both rutile [2,3] and anatase photocatalytic performances [4,5]. Observation that Sn-doping considerably improves photodegradation under visible light has become a further substantial step forward [3-6].

The positive effect of Sn on titania catalytic efficiency was attributed to a change of the anatase electronic structure - decrease of band gap (shift of absorption edge toward visible light area), and introduction of specific Sn-related surface sites was also mentioned [3,4,6]. Actually the studies on Sn-modification of titania discussed hypotheses that it can introduce heterojunctions improving a charge-hole separation [1], increase photo-excited electron–hole lifetime within a single particle [2] alter the electronic structure of the metal oxide photocatalysts [3,4,6] increase the particle size [2,3,7,8] and porosity [6], and control the actually developed crystal facets [9]. The authors of practically each study have developed their own interpretation of the positive effect of Sn – obviously the Sn doping effect is neither satisfactorily understood nor unequivocally experimentally described.

This work reports on a proprietary synthesis procedure to Sn-doped titania photocatalysts developed by Štengl and co-workers [10-12] in aim to allow their possible future industrial production. With the same motivation, we have recently developed an "urea route" [13,14]. The UV photocatalytic efficiency of the titania formed by the "urea route" [15] is substantially improved by chemical modifications [16-18]; currently this route is used in production of commercially available photoactive paint (ww.detoxycolor.cz/en/). Contrarily to that "one pot" syntheses, majority of previously published synthesis routes to titania photocatalysts are multi-step processes employing metal alkoxides as raw materials, non-aqueous solvents, and/or including crystallization step after chemical synthesis of precursors. The use of rather complex synthesis protocols can be substantiated in making thin films [4,5] or building nanoarchitectures [19], but it seems needless for the production of powders. There are indeed several synthesis ways to catalytically efficient titania powders without a need of metallo-organic compounds and organic solvents, but they usually require separate thermal [20] or hydrothermal crystallization steps [7-9] applied to poorly crystalline or amorphous intermediates from wet synthesis. In this paper, we reported on a "one-pot" thermal hydrolysis of titanium peroxo-complexes in the presence of Sn(II) or Sn(IV) chloride in aqueous solution. That "hydrogen peroxide route" is based on hydrolysis of Ti(IV) sulfate, treatment of the resulting gel by hydrogen peroxide, and thermal decomposition of so formed peroxo-titanate under reflux at ambient pressure producing directly the photocatalyst [10,11,18]. The resulting powders were tested in photobleaching of Orange II dye in aqueous slurry under irradiation at wavelengths of 365 nm and >400 nm using a home-made reactor [21]. The decomposition of soluble dyes [22] belongs now among the most widely used proxy methods (simple quantitative tests) to study titania photocatalysts, although its scientific value has recently been criticized [23]. In comparison with previously prepared photocatalytic materials based on TiO_2_ modified by molybdenum [11], iodine [17], or tungsten [18], the tin-doped titanium oxides exhibit a promising photocatalytic activity under activation by light >400 nm.

## Results and discussions

Rutile and cassiterite have the same crystal structure with space group of P 4/mnm. SnCl_4_ and SnCl_2_·2H_2_O during the thermal hydrolysis of titania peroxo-compounds also works as a mineralizers similarly as in hydrothermal synthesis [24,25]. The mineralizers are adsorbed on the surface of crystallites and if the adsorption has selectivity to crystal facets, they control the final morphology of crystallites [26]. If the adsorption has no selectivity, the adsorbed additives on the surface may retard the deposition of the product component to decrease the growth rate and consequently the reduction of the particle size. For example, rutile TiO_2_ can be formed with the addition of a rutile-directing mineralizer such as SnCl_2_, SnCl_4_, NH_4_Cl, NaCl or SnO_2_[27,28], presence of some carboxylic acids or SO_4_^2-^ promotes anatase crystallization [29,30] and HNO_3_, AlCl_3_.6H_2_O, *α*-Al_2_O_3_, Al(OH)_3_ and glycolic acid favor brookite formation [31,32]. SnO_2_ has same crystal structure with TiO_2_ rutile, therefore it was easily formed and adsorbed on the surface of TiO_2_ to retard the growth of TiO_2_ particles and enhance new nucleation. TiO_2_ in rutile form was achieved in the as precipitated stage by the addition of SnO_2_ as rutile forming nuclei and the amount of SnO_2_ directly influences the structure of resulting powders [33].

### Phase composition of catalysts

**Figure 1:**
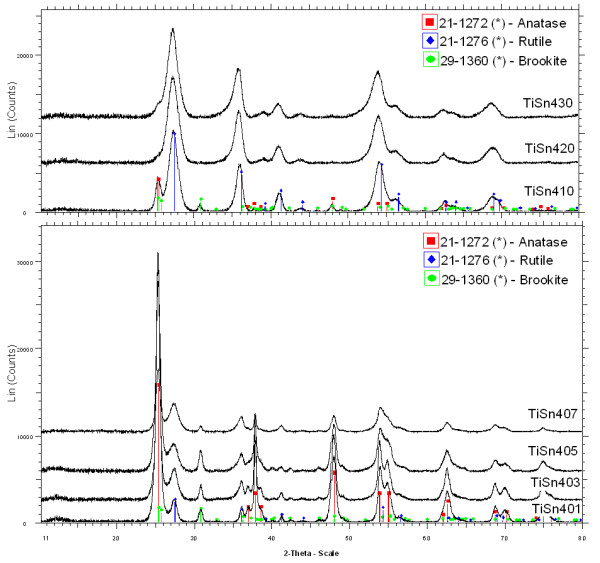
**The XRD patterns of the titanium oxides obtains in the presence of Sn**^**4+**^**.**

**Figure 2:**
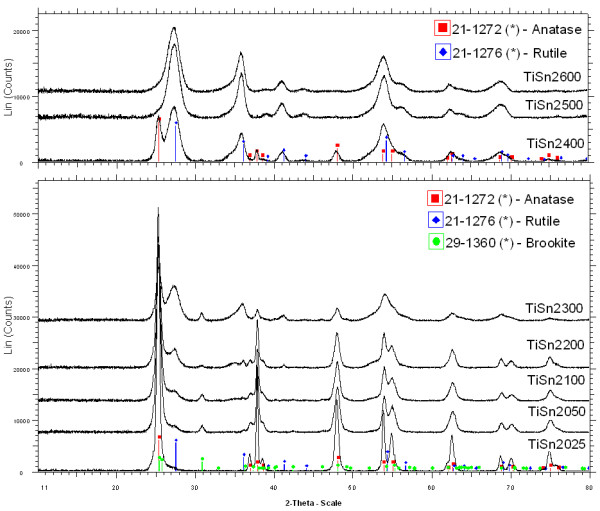
**The XRD patterns of the titanium oxides obtains in the presence of Sn**^**2+**^**.**

The XRD patterns of prepared Sn-doped titanium oxides are presented in Figures 1 and 2. A mixture of titania polymorphs containing anatase (ICDD PDF 21–1272), rutile (ICDD 21–1276), and/or brookite (ICDD 29–1360) were found in oxides obtained from mixtures with Sn ions, while only anatase was found at the lowest SnCl_2_ addition and only rutile at higher Sn addition. No diffraction lines of cassiterite (SnO_2_) were detected. The crystallite size and phase composition are listed in Tables 1 and 2. The cell parameters *a* and *c* of anatase and rutile and *a*, *b* and *c* of brookite were calculated by the Rietveld refinement and presented in Additional file 1: Tables S1 and S2. The products contain more rutile at lower Sn percentages if SnCl_4_ was used. These results are consistent with the rutile-promoting effect of Sn observed in wet synthesis [6] and wet synthesis followed by thermal [2,3] or hydrothermal [8] crystallization. Interestingly, hydrothermal (autoclave) recrystallization of wet synthesized mixed Ti-Sn precursors usually produced Sn-doped anatase [5,7]. Brookite formation in the absence of "phase-directing agents", chloride ions and strong acids is not common ([34] and references there).

**Table 1:** **Surface area, total pore volume, crystallite size and phase composition of Sn**^**4+**^**series of TiO**_**2**_

**Sample**	**SnCl**_**4**_**[ml]**	**Sn [wt.%]**	**Surface Area [m**^**2**^**g**^**-1**^**]**	**Total Pore Volume [cm**^**3**^**g**^**-1**^**]**	**Anatase by XRD [%]**	**Anatase Cryst. Size [nm]**	**Rutile by RD [%]**	**Rutile Cryst. Size [nm]**	**Brookite by XRD [%]**	**Brookite Cryst. Size [nm]**
TiSn401	0.1	0.71	96.5	0.32	84.1	46.2	11.2	43.3	4.7	30.1
TiSn403	0.3	1.03	101.5	0.33	75.5	47.8	18.5	25.7	6.0	47.0
TiSn405	0.5	1.97	121.1	0.27	65.5	49.2	19.3	18.9	15.2	36.7
TiSn407	0.7	3.45	140.0	0.32	46.1	32.5	50.4	16.1	3.5	34.4
TiSn410	1.0	5.04	157.7	0.23	5.1	-	88.5	15.9	6.4	24.3
TiSn420	2.0	9.30	194.6	0.20	-	-	100	15.6	-	-
TiSn430	3.0	12.27	185.9	0.21	-	-	100	10.2	-	-

**Table 2:** **Surface area, total pore volume, crystallite size and phase composition of Sn**^**2+**^**series of TiO**_**2**_

**Sample**	**SnCl**_**2**_**[g]**	**EDS Sn [wt.%]**	**Surface Area [m**^**2**^**g**^**-1**^**]**	**Total Pore Volume [cm**^**3**^**g**^**-1**^**]**	**Anatase by XRD [%]**	**Anatase Cryst. Size [nm]**	**Rutile by XRD [%]**	**Rutile Cryst. Size [nm]**	**Brookite by XRD [%]**	**Brookite Cryst. Size [nm]**
TiSn2025	0.25	1.0	128.9	0.59	100	49.0	0	-	0	-
TiSn2050	0.5	1.3	206.7	0.72	94.2	35.3	0.8	-	5.0	36.4
TiSn2100	1.0	3.6	158.8	0.51	91.2	35.0	3.8	-	5.0	35.8
TiSn2200	2.0	4.1	203.9	0.46	89.5	31.5	10.5	15.3	0	-
TiSn2300	3.0	8.3	254.6	0.44	42.5	29.6	50.2	17.3	7.3	34.6
TiSn2400	4.0	13.5	430.7	0.61	33.6	24.1	64.4	18.1	0	-
TiSn2500	5.0	18.1	366.9	0.37	0	-	100	19.8	0	-
TiSn2600	6.0	20.6	291.5	0.31	0	-	100	21.2	0	-

With increasing amount of Sn^4+^ there is a gradual diminishing of crystallites in all three phases (see Table 1). Concerned the structural properties, the very similar ionic radius of Ti^4+^ (0.605 Å) [35] and Sn^4+^ (0.690 Å) [36] would allow easy substitution of Ti^4+^ by Sn^4+^ in the TiO_2_ lattice, in comparison to Sn^2+^ that is much larger, 1.22 Å [37]. Therefore, Sn^2+^ can only occupy the interstitial sites in [TiO_4_ and [TiO_6_, resulting in a decrease in the lattice distortion energy, while Sn^4+^ can substitute for Ti^4+^ in [TiO_4_ and [TiO_6_, resulting in an increase in the distortion energy [38]. This paper supports those assumptions, the distortion of the lattice and increment the lattice parameters of Sn^4+^ doped TiO_2_ nanocrystals are demonstrated in Additional file 1: Table S2. The expansion of anatase, rutile and brookite cells attributes to the larger ionic radius of the doping Sn^4+^ ion than that of the lattice Ti^4+^ ion.

**Figure 3:**
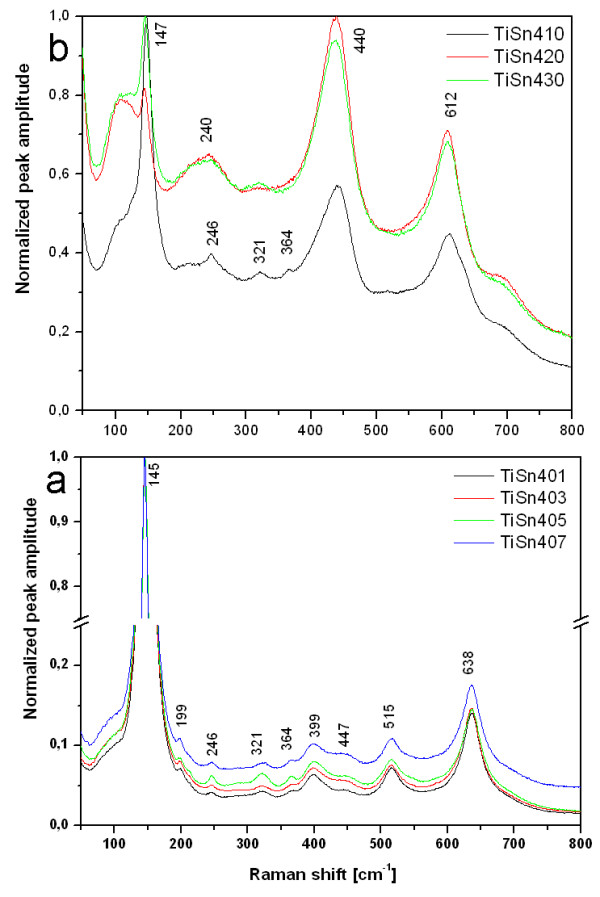
**The Raman spectra of the titanium oxides obtained in the presence of Sn**^**4+**^**.**

**Figure 4:**
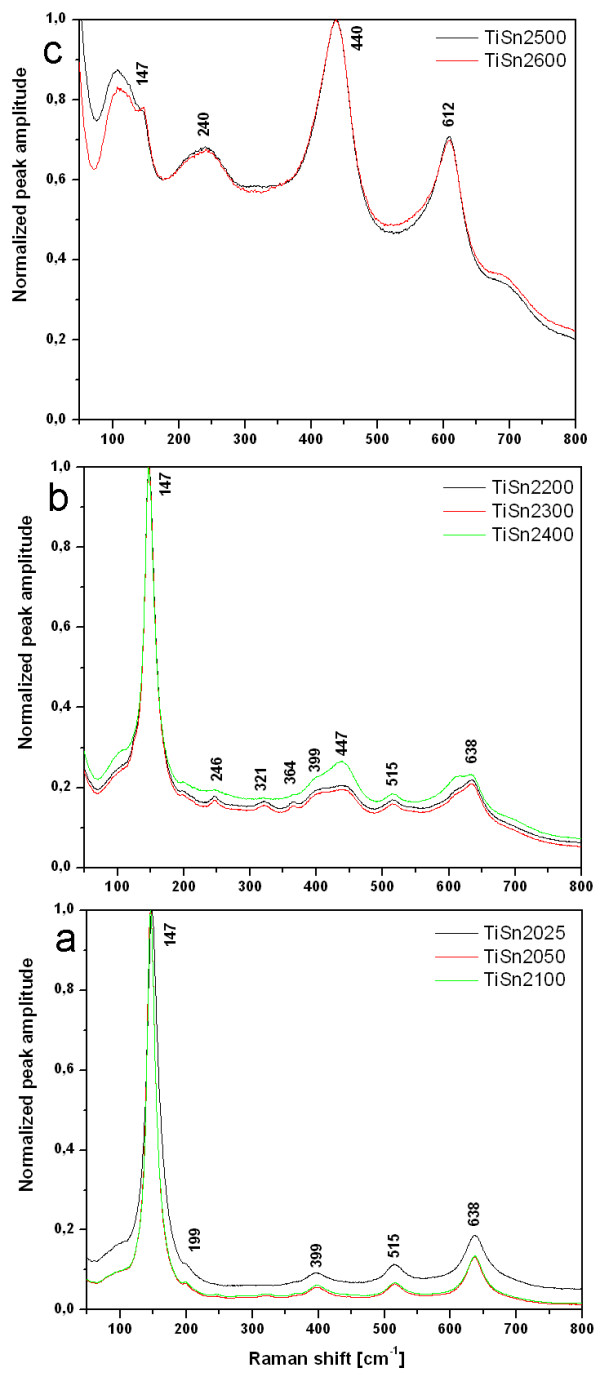
**The Raman spectra of the titanium oxides obtained in the presence of Sn**^**2+**^**.**

The phase composition obtained by XRD was confirmed by a Raman microanalysis. The Raman spectra of Sn^4+^- and Sn^2+^-doped titanium oxides are presented in Figures 3 and 4. The Raman bands were assigned to phases using published data [39-41]. Rutile TiO_2_ shows four Raman-active fundamental modes at 143 cm^-1^ (B_1g_), 447 cm^-1^ (E_g_), 612 cm^-1^ (A_1g_) , and 826 cm^-1^ (B_2g_) [39,41] and brookite have complex of 17 characteristic bands at 127, 154, 194, 247, 412, 640 cm^-1^ (A_1g_), 133, 159, 215, 320, 415, 502 cm^-1^ (B_1g_), 366, 395, 463, 584 cm^-1^ (B_2g_) and 452 cm^-1 ^(B_3g_) [40] of which only the most intense ones were registered. The specific vibration modes are located at 145 cm^-1^ (E_g_), 199 cm^-1^ (E_g_), 399 cm^-1^ (B_1g_), 515 cm^-1^ (B_1g_ + A_1g_) and 638 cm^-1^ (E_g_) indicating the presence of the anatase phase in samples denoted TiSn401, TiSn403, TiSn405 and TiSn407 [42]. The Raman band at 246, 321 and 364 cm^-1^ correspond with brookite phase and band at 448 cm^-1^ can be assigned to rutile. The samples TiSn401, TiSn402 and TiSn403 can be assigned to three of the four Raman rutile bands, namely at 147 cm^-1^(B_1g_), 440 cm^-1^ (E_g_) and 612 cm^-1^ (A_1g_). The series samples of Sn^2+^ doped TiO_2_ (see Figure 4) can be assigned to anatase (TiSn2025, TiSn2050 and TiSn2100), a mixture of anatase and brookite (TiSn2200, TiS2300, TiSn2400) or to pure rutile to samples TiSn2500 and TiSn2600. Moreover, the main Raman bands at 490, 574, 636, and 776 cm^-1^ attributed to SnO_2_ crystalline form were not detected, which indicates that tin does not exist as a separate crystalline oxide phase [43]. These results are in good agreement with those from the XRD analysis.

The samples TiSn401, TiSn403, TiSn405 and TiSn407 showed Raman shift from 145.3 to 150.6 cm^-1^ due to incorporation of Sn^4+^ to titania matrix. The Raman shifts cannot be affected by decreasing anatase crystallites size [44], as is evident from Table 1 – the titania crystals are too coarse to produce phonon confinement. In the TiSn2YY series the Raman band position of anatase is roughly constant with a ~ 147 cm^-1^.

A study by HRTEM with SAED was also consistent with the phase analysis. Diffraction line positions obtained by SAED are shifted to higher *d* with respect to pure anatase consistently with the results of the XRD study due to an anatase cell size expansion.

The Raman microscopy revealed a considerable difference in the homogeneity of the photocatalysts in the two series (SnCl_2_ and SnCl_4_) at a spatial scale of several μm (the spot size of the laser beam on the sample). The SnCl_2_ addition produced more uniform specimens, with only rare anatase-rich grains with a diameter <10 μm at higher Sn contents. The SnCl_4_ addition resulted in considerably more heterogeneous mixtures. TiSn403, TiSn405 and TiSn407 consist of an anatase matrix with small grains of an anatase-rutile mixture, while TiSn2050, TiSn2100 are quite homogeneous anatase-brookite mixtures at the μm scale. TiSn430 with the highest SnCl_4_ loading has a pure rutile matrix with occasional small grains of an anatase-rich anatase-rutile mixture. The total content of anatase in nearly pure rutile specimens is too small to be detected in the XRD patterns. The reason why Raman spectroscopy detected it is its higher sensitivity to anatase than rutile and also a microscopic setup of Raman analysis. Consequently traces of anatase were also revealed in the TiSn2500 and TiSn2600, in both cases as a minor admixture in the rutile matrix.

XRD lattice parameters of anatase and rutile obtained by the "hydrogen peroxide route" are larger than stoichiometric anatase and rutile, particularly *a* parameter of anatase and *a* parameter of rutile are expanded. Such small but definite lattice expansion was also found in the non-doped anatase obtained from titanium sulfate by the urea hydrolysis (*a* = 3.786 Å and *c* = 9.515 Å, [16]) as compared with pure anatase (*a* = 3.782 Å and *c* = 9.502 Å, [45]). The non-stoichiometry could be attributed to a cation deficiency presumably due to OH^-^ replacing O^2-^ excess of O ions (see the XPS results below).

Additional file 1: Figure S1 shows the IR spectrum of the Sn-doped TiO_2_ prepared by thermal hydrolysis of titanium peroxo-complexes. The broad absorption peaks at about 3400 cm^-1^ and the band at 1625 cm^-1^ correspond to the surface adsorbed water and the hydroxyl groups [46]. The band at 1385 cm^-1^ can be assigned to adsorbed carbonates on surfaces of TiO_2_, formed probably by the adsorption of CO_2_ from air [47]. Surface-adsorbed sulfate ions are probably responsible for a small band at 1100 cm^-1^[48]. The peak located at ~ 463 cm^-1^ in the FT-IR spectrum is likely due to the vibration of the Ti–O bond [49]. Since the Ti–O bond is shorter than the Sn–O bond, the doping of Sn^4+^ in TiO_2_ may lead to a shift of the lower wavenumber of Ti–O lattice vibration [50].

Obviously the titania formed by the "hydrogen peroxide route" without a subsequent hydrothermal or thermal treatment is not perfectly stoichiometric. A minor additional expansion of the lattice size of the anatase and rutile in Sn-modified titania is observed with a growing Sn percentage. This result is consistent with the previously published findings [2-4,8]. This Sn-specific lattice expansion is attributed to a larger ionic radius of Sn^4+^ (0.690 Å) than Ti^4+^ (0.605 Å) [35,51]. The lattice parameters of Sn-doped TiO_2_ nanocrystals are listed in Additional file 1: Table S2.

### XPS analysis

**Figure 5:**
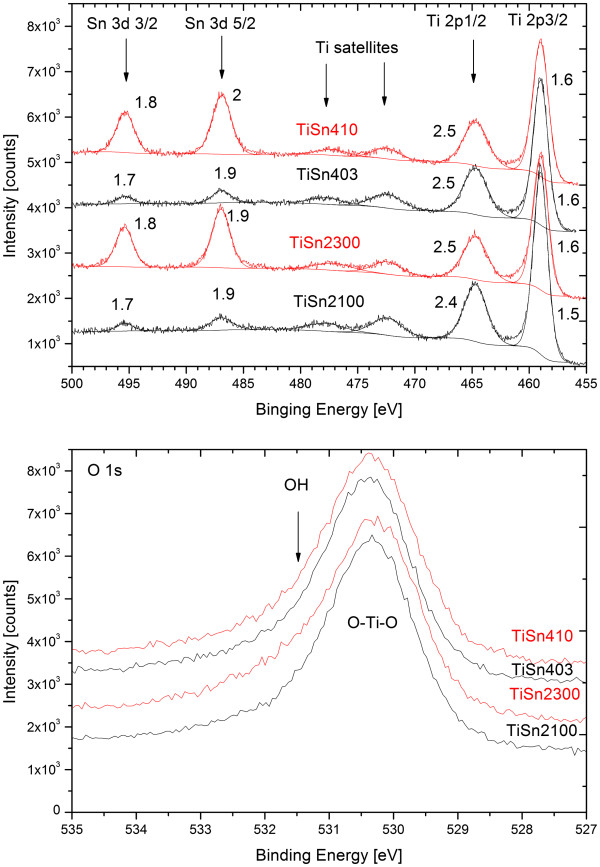
The selected regions from XPS spectra of Sn 3d, Ti 2p and O 1 s peaks on samples TiSn410, TiSn403, TiSn2300 and TiSn2100 with fitted peaks and indicated FWHM.

The chemical state of metal ions in TiSn2100, TiSn2300, TiSn403 and TiSn410 was evaluated from the high resolution XPS spectra (see Figure 5) of O 1 s, Sn 3d, C 1 s and Ti 2p. Because the ratio peak areas/RSF for Sn 3d and Ti 2p for each spin (spin-orbit coupling components) state were giving a little different concentrations Ti_1-x_(O,OH)_2_ (x > 0) therefore the ratio of both areas/RSF were always averaged. The compositions are summarized in the Additional file 1: Table S3. The O/Ti ratio show high oxygen over-stoichiometry. The high O/Ti ratio can partly be explained by the fact that the O1s spectra show a main peak at 530.3 eV with a shoulder at ~531.5 eV. The peak at 530.3 eV is assigned to the lattice oxygen, while the shoulder at 531.5 eV may be attributed to the oxygen in hydroxyl groups. These two distinct signals, denoted O_L_ and O_H_, were found in nearly equal concentrations in Sn-anatase obtained by hydrothermal synthesis [7] and in roughly 10% fraction of O_H_ after wet synthesis [6]. Obviously (at least) the surface of these titania specimens is strongly hydroxylated. The Sn/(Sn + Ti) ratios obtained by XPS (from the surface of the crystallites) and EDS (from the depth up to a few μm) are not significantly different in 3 samples of 4 analyzed (Additional file 1: Table S3). The catalysts obtained by the "hydrogen peroxide route" are hence not substantially surface enriched in Sn, as in most previous studies [3,6,7].

The high resolution measurements were made on Sn 3d and Ti 2p states. The Ti 2p3/2 and Ti 2p1/2 were identified at binding energies 459.0 eV and at 472.4 eV (472.3 eV in sample TiSn410). The Ti 2p3/2 state was used for the energy calibration in agreement with literature [52]. The control check was made on the binding energy of the C 1 s main component and the C 1 s was found at the binding energy about 285 eV, as expected for an ordinary surface contamination of samples handled under ambient conditions. The energy shifts due to the charging were similar to each other in all samples. The position of the Ti 2p peaks corresponds to the reported values and also the FWHM values about 1.6 eV for Ti 2p3/2 and 2.5 eV for Ti 2p1/2 are reasonable for single peak character, as is expected for Ti in TiO_2_. The control was made on the comparison of areas/RSF ratios for both spin states. The equal area/RFS ratios were found within precision about 10%. The limited precision is due to the background removal by the Shirley background. The peak positions and FWHM were found to be practically identical for all investigated samples. The two additional peaks at binding energies approximately 472.4 eV and approximately 477.8 eV are commonly attributed to plasmons originating from the Ti 2p peaks. The plasmon features are in our case shifted towards higher binding energies by 13.4 eV and 13.1 eV respectively (the second one is shifted by only 12.5 eV in the sample with a higher concentration of Sn) are known satellites from Ti 2p and they are connected to the existence of plasmons.

The satellites at the binding energy about 477.1 eV on the oxides with higher Sn contents are a bit closer to the main peak (12.5 eV) than reported in literature. It can influence the properties connected to the plasmons. The area of those satellites is about 20% of the main corresponding Ti peaks, which is close to 24% or 25% reported in literature [52]. The second satellite peaks were also reported with the binding energy shifts about 25.4 eV and 25.0 eV for Ti 2p3/2 and Ti 2p1/2, respectively [52]. The tin related peaks Sn 3d3/2 at 495.4 eV and Sn 3d5/2 at 486.7 eV were found in all samples and the corresponding FWHM were also practically identical.

Regretfully, the available literature values of binding energies of the Sn3d5/2 core levels for SnO (Eb = 487.1 eV [53], 487.4 eV [54]) and SnO_2_ (Eb = 487.01 eV [55], 486.8 eV [56]) nearly coincide, which made it hardly possible to differentiate between SnO and SnO_2_. On the other hand, in the above mentioned literature when the set of similar samples with SnO or SnO_2_ was measured and evaluated collectively, the differences between both states were clearly visible. The SnO_2_ should have a higher binding energy than SnO by about 0.5 eV and hence in the SnO + SnO_2_ mixtures the peak should be relatively broad [57]. This broadening has to be observed in both spin states of Sn 3d peaks. These peaks in all samples have clearly single peak character with FWHM <2 eV. It is, of course, possible to use two components for such deconvolution, but then the FWHM would not be realistic - they would be either too narrow or one of the peaks would have only a very small area. Therefore the presence of mainly more stable SnO_2_ in the samples is most probable, while SnO phase can still be present in concentrations by an order of magnitude lower. Additionally, the small broadening of the peaks on the lower binding energy side can also be caused by the second satellite peak of Ti 2p [52] expected at binding energy about 484 eV or background subtraction process or finally by a signal noise. This evaluation is in agreement with the expectation that Sn^2+^ should be quickly oxidized during the synthesis and subsequent sample handling in air.

The control check was made by the comparison of areas/RSF for both spin-orbit coupling components and the area/RSF ratios were found the same with relative deviations below 7% for samples TiSn410 and TiSn2300 with higher Sn concentrations. The other specimens with low Sn concentrations have a poor agreement between the area/RSF ratios and the relative deviations were about 16% for sample TiSn403 and 29% for sample TiSn2100. The poor precisions here are probably due to the background removal process from these noisy spectra. All the binding energies and FWHM values are summarized in Additional file 1: Table S4.

### Particle size and shape, porosity

With increasing amount of Sn^4+^ there is a gradual decrease of a mean coherence length in all three titania phases as well as an increase of the total specific surface areas (see Table 1), which indicates that Sn presence decreases the growth rate of titanium oxide crystals. That inhibiting effect of Sn on the titania crystal growth is well known [2,3,7,8]. More interesting is that with increasing amount Sn^4+^ and Sn^2+^ there is a change of the pore size distribution: pores from the range of 10–20 nm gradually shifts to the size between mesopores and micropores (~ 3 nm). The Barrett-Joyner-Halenda (BJH) pore-size distribution plot and nitrogen adsorption/desorption isotherms of as-prepared Sn-doped TiO_2_ are shown in Additional file 1: Figures S2 and S3. All samples have a type IV isotherm, which is characteristic of large-pore mesoporous material with a H2 type hysteresis. The high steepness of the hysteresis indicates a high order of mesoporosity. All samples are characteristic of Type A hysteresis loop according to de Boer's characterization [58] attributed to capillary pores - tubes open at both ends, wide ink-bottle pores and wedge-shaped capillaries.

**Figure 6:**
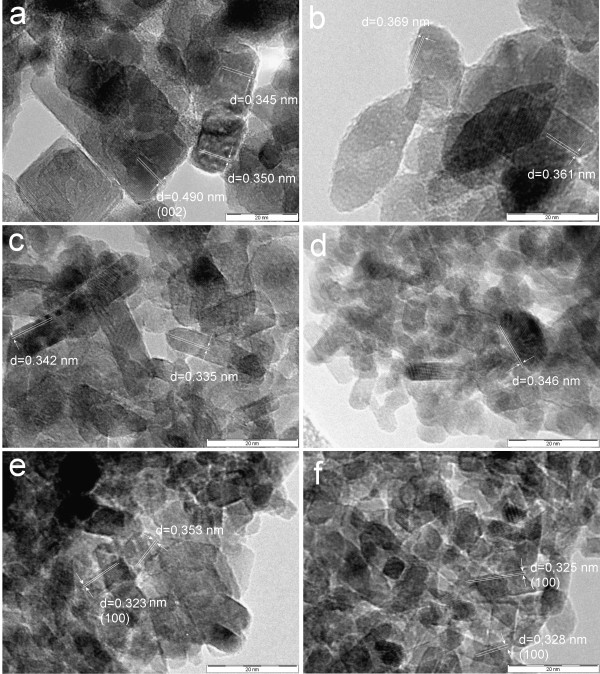
**The TEM images of Sn**^**4+**^**-doped titanium oxides: a) TiSn401, b) TiSn405, c) TiSn407, d) TiSn410, e) TiSn420 and f) TiSn430.**

**Figure 7:**
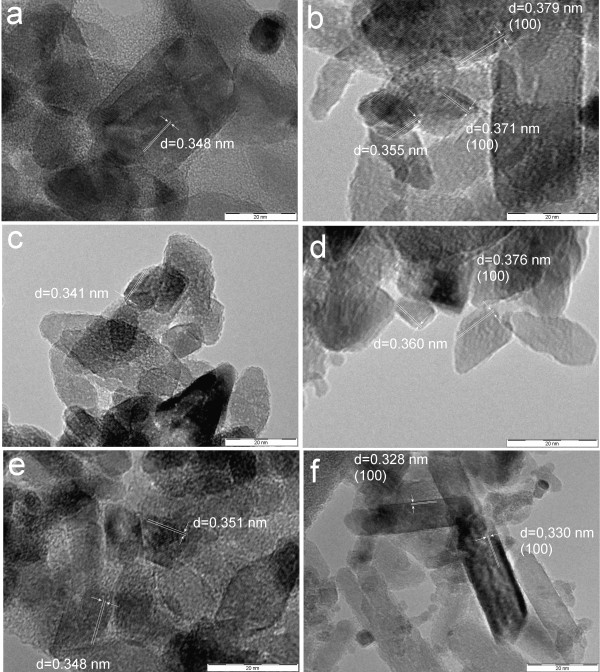
**The TEM images of titanium oxides obtained with Sn**^**2+**^**: a) TiSn2050, b) TiSn2100, c) TiSn2200, d) TiSn2300, e) TiSn2400 and f) TiSn2600.**

**Figure 8:**
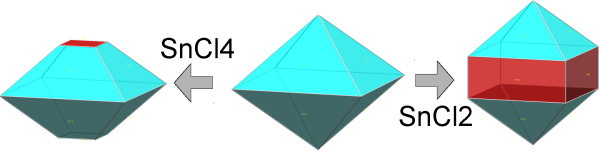
Changes of crystal morphology of anatase.

TEM images of Sn-doped titanium oxide crystals are shown in Figures 6 and 7. The interlayer spacing *d* ~ 0.34 - 0.36 nm in micrographs of Sn401, Sn405 and Sn420 can be assigned to crystal plane (101) of anatase or (120) of brookite (Figures 6a, b, e), interlayer spacings *d* = 0.342 and 0.346 nm in micrographs Sn407, Sn410 (Figures 6c, d) evidently correspond to crystal planes (111) of brookite. The samples Sn420 and Sn430 have interlayer spacing *d* ~ 0.32-0.33 nm, which are from crystal planes (100) of rutile (Figures 6e, f). The crystal planes *d*_002_ = 0.49 nm in sample TiSn401 (Figure 6a) indicate a change in the anatase crystal morphology, as can be seen in Figure 8.

The interlayer spacing shown in Figures 7a, b, c have *d* in the range 0.346 - 0.360 nm and corresponds to crystal planes (101) anatase or (120) of brookite. The presence of lattice spacing *d*_100_ = 0.38 nm for the (100) anatase plane in TiSn2300 and TiSn2100 (Figures 7b, d) may indicate the occurrence of (010) anatase plane that is perpendicular to the [010] direction [59]. The incorporation of Sn to titania leads to an increase in the area of (010) surfaces at the expense of (101) and (10–1) and thus changes the morphology of the anatase crystal similarly as incorporation of W [17]. The interlayer spacing ~ 0.331 nm corresponds to crystal surfaces (100) of rutile (Figure 7f). Changes of the anatase crystal morphology resulting in increasing crystal plane (010) are shown in Figure 8. The selected area electron diffraction patterns (SAED), presented in Additional file 1: Figures S4 and S5, were analyzed by the Process Diffraction program that confirmed the structure composition results from XRD analysis. The Sn doping hence affects the formation of new crystal planes, which allow origin of next crystal facets, leading to a change in morphology of anatase (Figure 8). The crystal habit, i.e., the actual crystal facets evolved, can markedly change the specific activity of the titania catalysts [9,12,60-63].

### UV/Vis absorption spectra

Additional file 1: Figure S6 presents UV/Vis absorption spectra of the as-prepared tin-doped photocatalysts. Compared with the non-doped titania, the absorption edge of the doped samples TiSn401, TiSn403, TiSn405, TiSn407 and TiSn410 are red-shifted. The largest shift was found for a sample TiSn410, of which absorption tail extends to ~ 425 nm (Additional file 1: Figure S6a). Samples TiSn420 and TiSn430 have an adsorption edge corresponding to the rutile phase (~ 410 nm). The photocatalysts in TiSn2YYY series are red-shifted proportionally to the tin concentration (see Additional file 1: Figure S6b). TiSn2YY at higher Sn loadings and all TiSn4XX have additionally measurable absorption (f(R) = 0.02 - 0.05 Kubelka-Munk units) in green to red region (> 500 nm). Both the red-shifted absorption edge and the novel bands in Vis could be responsible for the photoactivity under >400 nm light. Also previous researchers found that Sn-doping in anatase shifts the absorption band edge to longer wavelengths [3,6,7] and at higher loading it produces new bands absorbing roughly between 400 and 500 nm [3,5,6], probably as a consequence of formation of new electron levels of Sn ions in the titania band structure.

Additional file 1: Figure S7 shows the (A hν)^2^ versus photon energy for a direct band-gap transition. The value of 3.20 eV is for un-doped titania denoted as TiSn000. The value of band-gap energy decreases with increasing Sn content. The band gap of bulk SnO_2_ is 2.6 eV, which corresponds to an absorption edge of ~450 nm. The sample denoted TiSn410 has the lowest E_bg_, next samples TiSn420 and TiSn430 with pure rutile phase have E_bg_ corresponding to rutile and incorporation of Sn^4+^ ions apparently does not affect the E_bg._ Conversely for samples obtained in the presence of Sn^2+^ ions there is an evident shift of E_bg_ up to the value of ~ 2.95 eV.

### Photobleaching of Orange II dye

The photocatalytic activity of the titanium oxides was tested by photobleaching of 5 mmol Orange II dye aqueous solutions under UV radiation at 365 nm (UV-A, "Black Light") and >400 nm ("Warm White" lamp by Narva, its spectrum is shown in [64]). Photobleaching of soluble dyes is a commonly used test of novel catalysts [1,6,22] irrespective of a complex nature of that reaction with some mechanistic aspects unclear [23].

According to the degradation pathway proposed by Zhao et al. [65], the main byproducts formed by the ozonation of azo-dye are organic acids, aldehydes, ketones, and carbon dioxide. Demirev and Nenov [66] suggested that the eventual degradation products of azo dye in the ozonation system would be acetic, formic and oxalic acids. The reaction pathway for the visible light-driven photocatalytic degradation of Orange II dye in aqueous TiO_2_ suspensions is schematically shown in [67].

On kinetics of heterogeneous photocatalysis for decomposition of model compounds such as dyes Orange II Langmiur–Hinshelwood equation [68] can be used:

(1) $$r=-\text{d[OII]/dt}={-k\cdot }_{r}\text{·K[OII]/}(1+\text{K·[OII}])$$

where r is the degree of dye mineralization, k_r_ the rate constant, t the illumination time, K is the adsorption coefficient of the dye and [OII] is dye concentration. At very low concentration of the dye, in the validity of Lambert-Beer Law [69]:

(2) A=ε · c · 1

where *A* is the absorbance, *c* the dye concentration, *l* the length of absorbent layer and ε is the molar absorption coefficient, it is possible to simplify equation (1) to the first order kinetic equation:

(3) $$\ln\left({\left[\text{OII}\right]}_{o}\right)+{\text{K([OII]-[OII}]}_{o})=-k_{r}\cdot k\cdot t$$

and after integration:

(4) $$\left[\text{OII}\right]\;=\;{\left[\text{OII}\right]}_{o}\cdot \text{exp(-k·t);}\left(k=k_{r}\cdot k\right)$$

For samples of Sn doped titania at photocatalytic degradation under visible light different course of reaction has been observed. The kinetic curves showed a faster progress in its beginning.

The slowing of total photocatalytic decomposition may be due to heterogeneity of the sample, i.e. different rates of decomposition in various TiO_2_ crystalline modifications, anatase, brookite and rutile, or insufficient degradation of intermediates of photocatalytic reaction. Therefor the equation (5) was adapted to the following form:

[OII] = [OII]_1_·EXP(−k_1_·t) + [OII]_2_·EXP(−k_2_·t); [OII]_0_ = [OII]_1_ + [OII]_2_ + [OII]_3_ (5) where [OII]_0_ is initial concentration of dye, [OII]_1_ is concentration of dye in time t, [OII]_2_ is concentration of reaction intermediates of photocatalytic degradation and [OII]_3_ is non-degradable residuum. In case if the intermediates decompose fast enough, then k_1_ = k_2_ and the equation (5) goes to form (5).

**Figure 9:**
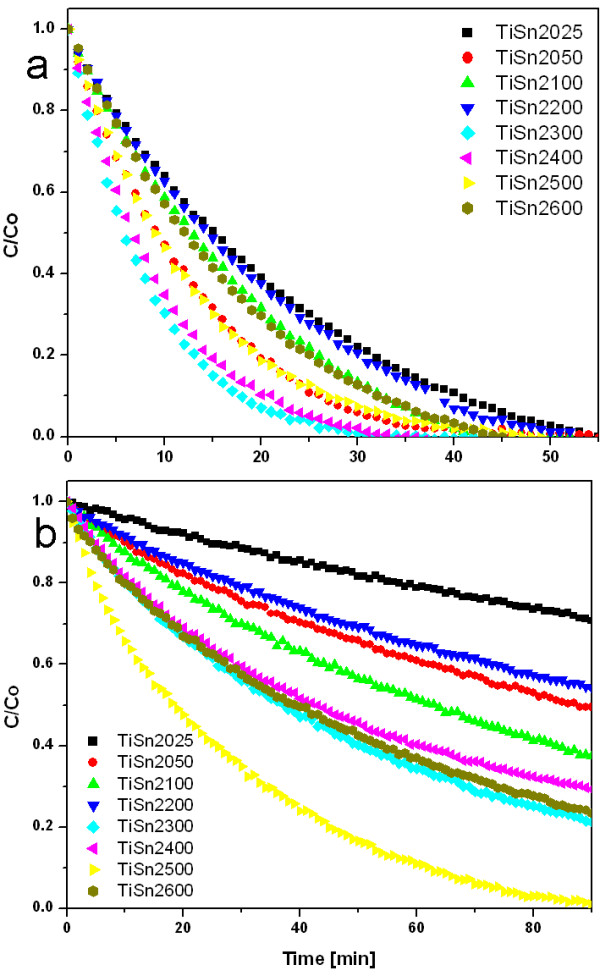
**Orange II dye photobleaching on titanium oxides obtained with Sn**^**2+**^**at wavelength a) 365 nm and b) 400 nm.**

**Figure 10:**
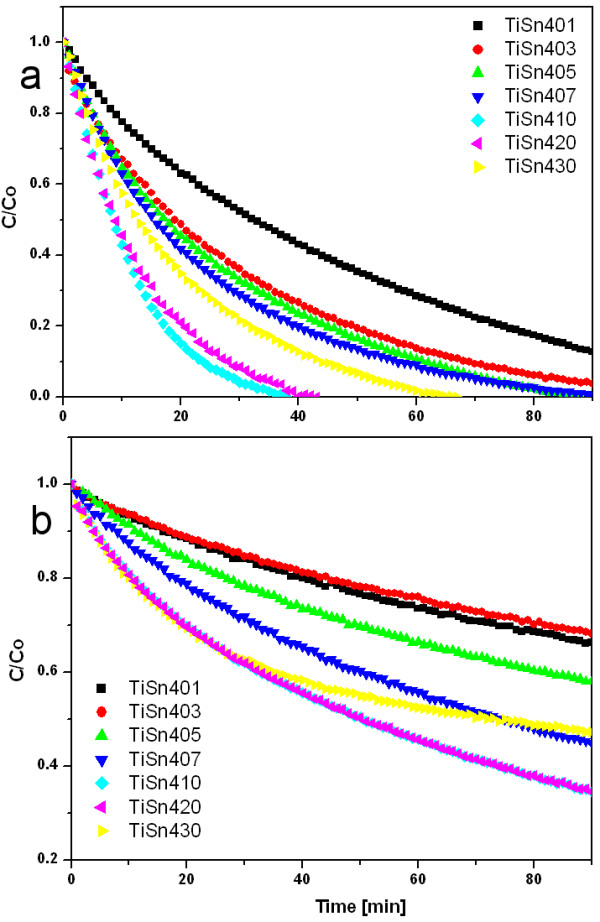
**Orange II dye photobleaching on titanium oxides obtained with Sn**^**4+**^**at wavelength a) 365 nm and b) 400 nm.**

The calculated degradation rate constants k for a reaction following first order model kinetics or k_1_ and k_2_ (min^-1^) respectively, of degradation of Orange II dye at 365 nm (Black light) and 400 nm (“Warm White” light) are shown in Additional file 1: Table S5 and kinetics of degradation are presented in Figures 9 and 10.

The specific surface area of the catalysts increases with the increasing Sn content as a consequence of the presence of brookite and growing percentage of rutile with smaller particles than anatase; also decreasing particle size of anatase contributes to the surface area growth. As a consequence of all these phenomena, the specific surface area σ(BET) of the catalysts is simply directly proportional to the total Sn content:

(5) $$\sigma \left(\text{BET}\right)\;=\;11.9\cdot \;\text{Sn}\;(\text{wt}.\%)\;+\;120,r2=0.6372$$

irrespectively of the form of Sn modification. To decipher the increase of the total available surface area of the catalyst and a possible specific effect of the Sn species, the rate coefficients were divided by the specific surface area of the catalysts for further data evaluation. For the reaction under UV irradiation there is a flat maximum of the specific reaction rate coefficient at Sn content between 4 and 10 wt.%, irrespective of whether SnCl_2_ or SnCl_4_ was used for the synthesis. The samples marked TiSn410 and TiSn2300, which contains ~5 wt.% of Sn and ~8 wt.% Sn show the highest photocatalytic activity.

The catalytic activity of the SnCl_2_ series under >400 nm light is much lower than activity of the SnCl_4_ series, although both phase composition and particle size of the phase constituents in these two series are similar. The reaction rate coefficients normalized to the specific surface areas have a maximum at Sn contents between 0.7 and 5 wt.% in the SnCl_4_ series, in which the Sn doping has an effect of remarkable "onset" of activity, then it is only weakly dependent on the actual Sn content: the surface-area normalized rate coefficients increased 25 times with respect to the pure titania. In the SnCl_2_ series the impact of Sn-modification is much less substantial: the Sn-doping increases the surface-area normalized rate coefficients only up to 5 times. This remarkable difference between the two series offers an opportunity to reveal the properties or features relevant for the photobleaching activity under >400 nm light.

Two substantial differences between the SnCl_2_ and SnCl_4_ series are 1) heterogeneity of the phase composition and 2) Vis absorption features in the UV/Vis spectra. SnCl_4_ series is considerably more heterogeneous in terms of a bulk phase composition - most specimens contain all three titania polymorphs and additionally there is a considerable heterogeneity at μm spatial scale revealed by the Raman microanalysis (above). This apparent beneficial effect of heterogeneity at an intimate level of crystal contacts points to the seminal study by Vinodgopal and Kamat [1] on photocatalysis by physical mixtures of colloidal SnO_2_ and TiO_2_. There is no "tail" in the UV/Vis spectra of the Sn-doped titania in the region 400–500 nm, but all these specimens have a distinct long-wavelength absorption (>500 nm). Probably the red shift of the absorption edge is not responsible for the catalytic activity improvement by Sn, otherwise SnCl_2_ series would have been more active than SnCl_4_ series.

### Evaluation of Sn-titania obtained by "hydrogen peroxide route"

There is no universal way to compare catalytic efficiency of titania specimens from published data, because practically each laboratory uses a different catalytic test. It is hence possible to evaluate only actual improvement in a given series of catalysts due to a given chemical modification to verify its real impact. Ohtani [23] correctly pointed to the lack of understanding of the detailed reaction mechanism of the azo dye photobleaching, but it still remains the simplest empirical way to handle large sample series with a reasonable effort and produces very useful insight into the measurable effect of the titania doping. For the catalysis under UV irradiation, novel materials are commonly compared with Degussa P25 titania, but for the Vis light there is no similar "reference". In our present study, the most efficient Sn-modified titania specimens obtained by the "hydrogen peroxide route" have the rate coefficients of the Orange II photobleaching under >400 nm light by more than ten times larger initial rate than a control, non-doped titania. This is comparable to the best relative improvement yet reported for the Sn-modification [6]. Oropeza et al. [3] found that Sn-doping caused an onset of photocatalytic activity under >420 nm irradiation in the comparison with the non-doped commercial titania catalysts and a better performance of the Sn-doped rutile than the N-doped titania. On the other hand, Cao et al. [4] and Zhao et al. [7] have found much less substantial effect of Sn (twofold increase in the rate coefficient). In the reports [70], [71] the effect of Sn addition was even smaller – obviously the Sn-modification is fully utilized only under certain synthesis conditions, actually including also our "hydrogen peroxide route". The best photocatalytic activity under >400 nm light was achieved with the Sn^4+^-doped sample TiSn410, which has the rate coefficients k_1_ = 0.0834 min^-1^ and k_2_ = 0.00926 min^-1^. This result is comparable to or better than the best catalysts from the titania series obtained by modification using Mo [11], W [12], I [17], N [72] and B [72,73], which were also subjected to the same kinetic test as in this present study, the Orange II photobleaching at the same experimental setup.

The activity of the Sn-modified titania under the UV light can be compared with several previous titania specimens analyzed by the same kinetic test. The samples marked TiSn410 and TiSn2300, which contains ~5 wt.% of Sn^4+^ and ~8 wt.% of Sn^2+^, show the highest photocatalytic activity with the rate coefficients 0.0942 min^-1^ and 0.126 min^-1^, respectively, under UV light. The photobleaching by the tin-doped titanium oxides under UV is hence more than twice faster than by the Mo-doped titania (k = 0.0461 min^-1^) [11], W-doped titania (k = 0.0617 min^-1^) [12] and nearly the same as the I-doped titania (k = 0.0851 min^-1^) [17] prepared with the same synthesis method (the "hydrogen peroxide route"). The achieved reaction rate with Sn-doped titanium oxides is more than twice faster than with P25 under the same kinetic test (k = 0.047 min^-1^) [16].

### Experimental

#### Synthesis of tin-doped titanium oxides

All chemicals used, titanium oxo-sulfate (TiOSO_4_), tin(IV) chloride (SnCl_4_), tin(II) chloride (SnCl_2_·2H_2_O) ammonium hydroxide (NH_4_OH) and hydrogen peroxide (H_2_O_2_) were of an analytical grade and were supplied by Sigma–Aldrich Ltd.

In a typical experiment, 100 mL of a 1.2 M stock solution of titanium oxo-sulfate was diluted in 1 L of distilled water and hydrolyzed by slow addition of ammonium hydroxide solution (10%) under continuous stirring at temperature of 0°C in ice bath, until the reaction mixture reached pH 8.0.

At 0 °C does not proceed anatase or rutile nucleation and reaction resulted in amorphous phase Ti(OH)_4_, which can be easily dissolved by H_2_O_2_ to obtain right solution of titania peroxo complex, which gradually resulted to yellow gel. Precipitation above 0°C leads to formation of rutile or anatase nuclei and insufficient dissolution in titania peroxo-complex.

The obtained white precipitate was separated by filtration and washed with distilled water until it was free of sulfate ions (checked by the BaCl_2_ test). It was then mixed with 100 mL of 30% hydrogen peroxide solution that resulted in a homogeneous and transparent yellow gelatinous mass. That mass was subsequently mixed with defined amount of liquid tin(IV)chloride (SnCl_4_) dosed with syringe, or solid tin(II) chloride (SnCl_2_·2H_2_O); the actual ratios of the raw materials are listed in Tables 1 and 2. The reaction mixture was then refluxed in a round-bottom flask in a heating mantle at 100°C. On heating, the yellow gelatinous mass first turned dark orange and then it was gradually dissolved. During prolonged heating the solution color weakened and a yellowish white precipitate was formed. The refluxing was continued till the precipitate turned white, which lasted for ~ 36 hours. The obtained doped titanium oxides were air dried at 105°C. Seven Sn^4+^-doped titanium oxides were obtained and denoted TiSn4XX, where XX is the input volume of SnCl_4_ (in ml) and eight specimens obtained with Sn^2+^ were prepared and denoted TiSn2YYY, where YYY is the input weight of SnCl_2_·2H_2_O (in grams).

## Conclusions

The "hydrogen peroxide route" is an efficient and simple synthesis ("one-pot") method leading to Sn-modified titania photocatalysts remarkably active in the Orange II photobleaching under >400 nm light. The most active catalysts were those consisting of two or three titania polymorphs, mainly anatase and rutile with minor brookite admixture. The Sn content decreases the particle size of titania polymorphs and increases the specific surface area of the catalysts, which is obviously beneficial for the photobleaching rate coefficients. The onset of the activity under >400 nm light was particularly significant if SnCl_4_ was used in the synthesis; in that series more rutile was formed and the products had a more heterogeneous phase composition on the μm scale level. The formation of Sn-doped rutile and/or its presence in the catalyst seem to be responsible for the beneficial effect of the Sn doping. Additional feature specific to the titania specimens formed by the "hydrogen peroxide route" and possibly contributing to their improved activity is the oxygen over-stoichiometry due to the presence of OH^-^ groups replacing O^2-^ groups of the titania. UV/Vis spectra of the most active catalysts had their absorption edges slightly red shifted and exhibited novel absorption bands at >500 nm. Both the used synthesis route and the Sn modification are promising ways to cheap and efficient photocatalysts for the Vis light activation.

## Methods

### Characterization and analysis of solids

Diffraction patterns were collected with diffractometer PANalytical XÂÂ´Pert PRO with Cu X-ray tube and PIXcel detector [74]. A qualitative analysis was performed with the DiffracPlus Eva software package (Bruker AXS, Germany) using the JCPDS PDF-2 database [75]. For a quantitative analysis of XRD patterns we used Diffrac-Plus Topas (Bruker AXS, Germany, version 4.1) with structural models based on ICSD database [76]. This program permits to estimate the weight fractions of crystalline phases and mean coherence lengths by the Rietveld refinement.

The element composition of the catalysts was obtained by a conventional SEM/EDS analysis. The particle morphology was inspected by a transmission electron microscopy (TEM) and the crystal structure was analyzed by an electron diffraction using a 200 kV TEM microscope JEOL 2010 F. A microscopic copper grid covered by a thin transparent carbon film was used as a specimen support. The photocatalysts were studied in a bright field and by the electron diffraction with a selecting aperture (SAED) mode at an acceleration voltage of 200 kV.

The surface areas of the photocatalysts were determined from nitrogen adsorption–desorption isotherms at a liquid nitrogen temperature using a Coulter SA3100 instrument with outgas 15 min at 150°C. The Brunauer–Emmett–Teller (BET) method was used for the surface area calculation [77], the pore size distribution (pore diameter, pore volume and micropore surface area of the titanium oxides) was determined by the Barrett–Joyner–Halenda (BJH) method [78].

The Raman spectra were acquired with a DXR Raman microscope (Thermo Scientific) with 532 nm laser; 32 two-second scans were accumulated with 532 nm laser (3–4 mW) under 20-50x objective of an Olympus microscope. An analyzed spot size was 1–2 μm. Infrared spectra were recorded by a Thermo-Nicolet Nexus 670 FT-IR spectrometer approximately in 4000–500 and 500–50 cm^-1^ with single-reflection horizontal accessory on Si crystal. The photocatalysts were mixed with KBr and pressed into pellets at ambient conditions and measured in the transmission mode.

An XPS apparatus was equipped with SPECS X-Ray XR50 (Al cathode 1486.6 eV) and a SPECS PHOIBOS 100 Hemispheric Analyzer with a 5-channel detector. A background pressure during the measurements was < 2·10^-8^ mbar. XPS survey-scan spectra were made at pass energy of 40 eV; the energy resolution was set to 0.5 eV, while individual high-resolution spectra were taken at pass energy of 10 eV with 0.05 eV energy steps. A software tool CasaXPS was used to fit high-resolution multi-component peaks. The proper surface-charge compensation was done by fitting C-C, C-H component of C 1 s peak to reference binding energy 284.5 eV. The atomic concentration of compounds was evaluated with relative sensitivity factors (RSF) defined in a standard table of the CasaXPS software.

A Perkin Elmer Lambda 35 spectrometer equipped with a Labsphere RSAPE- 20 integration sphere with BaSO_4_ as a standard was used for the diffuse reflectance UV/Vis spectra. The spectra were recorded in the diffuse reflectance mode and transformed to absorption spectra through the Kubelka-Munk function [64,79]:

(6) $$\text{f(R})\;=\;{\left(1-r\right)}^{2}\text{/ 2R}$$

where f(R) is absorbance and R is the reflectance of an "infinitely thick" layer of the solid.

### Photocatalytic activity test

Photocatalytic activity of samples was assessed from the photobleaching kinetics of Orange II dye (sodium salt 4-[(2-hydroxy-1-naphtenyl)azo]-benzene sulfonic acid) in 1000 ml of aqueous slurries using a self-constructed photoreactor [21]. It consists of a stainless steel cover and a quartz tube with a fluorescent lamp by Narva with power 13 W and a light intensity ~ 3.5 mWcm^2^ (commercial name "Black Light", 365 nm) and a Narva lamp with commercial name "Warm White" (emission spectrum >400 nm). The emission spectra of both light sources were shown in [73]. 0.5 g photocatalyst was dispersed for 10 min in an ultrasonic bath (300 W, 35 kHz) before use; actually that dispersion plays a crucial role in obtaining reproducible results of the kinetic tests. The pH of the resulting suspension was taken as the initial value for neutral conditions and under the experiment it was kept at a value of 7.0. Orange II dye solution was circulated by means of a membrane pump through a flow cell. The concentration of Orange II dye in the suspension was determined by measuring absorbance at 480 nm with Vis spectrophotometer ColorQuestXE. The suspension contained 5 mmol of the dye at the beginning of the kinetic test, which is a substantial excess over what can be absorbed by the catalyst. Maximal adsorption of structurally similar azo dyes Orange G and Methyl Orange is < 10 μmol per gram of P25 titania [22], hence in our experimental setup the azo dye amount exceeds the titania adsorption capacity by about two orders of magnitude. None of the two used light sources can degrade Orange II without a photocatalyst. The kinetic experiment started by switching on the light source after the spectral signal of the Orange II in the suspension reached a steady state; this actual initial signal was taken as a measure of the initial concentration of the dye. The sorption of the dye on the catalysts is hence irrelevant for the evaluation of the kinetic experiments.

## Competing interests

The authors declare that they have no competing interests.

## Authors’ contributions

VŠ was the main architect of this paper; he was responsible for the synthesis, catalytic tests and range of analytical methods. TMG performed Raman spectroscopy and contributed to the data interpretation. JH performed a lot of lab work and technical help with the manuscript. MK performed XPS analyses and most of their interpretations. All authors read and approved the final manuscript.

## Supplementary Material

Additional file 1 **Table S1.** Cell parameters *a*, *b* and *c* of anatase, rutile and brookite doped with SnCl2. **Table S2.** Cell parameters a, b and c of anatase, rutile and brookite doped with SnCl4. **Figure S1.** Infrared spectra of series samples a) Sn4+ doped TiO2 and b) Sn2+ doped TiO2. **Table S3.** Atomic concentrations of elements from XPS measurements. **Table S4.** Binding energies and FWHM of Sn 3d and Ti 2p peaks. **Figure S2.** Pore area distribution of a) TiSn2025, b) TiSn2100, c) TiSn2200, d) TiSn2300, e) TiSn2500 and f) TiSn2600. Inset are hysteresis loops. **Figure S3.** Pore area distribution of a) TiSn401, b) TiSn403, c) TiSn405, d) TiSn410, e) TiSn420 and f) TiSn430. Inset are hysteresis loops. **Figure S4.** Selected Area Electron Diffraction (SAED) of sample a) TiSn2050 - anatase, b) TiSn2100 - anatase, c) TiSn2200 - anatase, d) TiSn2300 - brookite, e) TiSn2400 - anatase and f) TiSn2600 - rutile. **Figure S5.** Selected Area Electron Diffraction (SAED) of sample a) TiSn401 - anatase, b) TiSn405 - anatase, c) TiSn407 - anatase and brookite, d) TiSn410 - rutile, e) TiSn420 - rutile and f) TiSn430 - rutile. **Figure S6.** UV-VIS spectra of series samples a) Sn2+ doped TiO2 and b) Sn4+ doped TiO2. **Figure S7.** Band-gap energy of titanium oxides prepared in the presence of a) Sn2+ and b) Sn4+. **Table S5.** Rate constant k k, k1 and k2 of tin doped titania. Click here for file
