# Supplementary material for: Hydrogen peroxide route to Sn-doped titania photocatalysts
Source: Chem Cent J. 2012 Oct 5;6:113. doi: 10.1186/1752-153X-6-113 (PMC3531300; doi:10.1186/1752-153X-6-113)
Supplement: Additional file 1 — Table S1. Cell parameters a, b and c of anatase, rutile and brookite doped with SnCl2. Table S2. Cell parameters a, b and c of anatase, rutile and brookite doped with SnCl4. Figure S1. Infrared spectra of series samples a) Sn4+ doped TiO2 and b) Sn2+ doped TiO2. Table S3. Atomic concentrations of elements from XPS measurements. Table S4. Binding energies and FWHM of Sn 3d and Ti 2p peaks. Figure S2. Pore area distribution of a) TiSn2025, b) TiSn2100, c) TiSn2200, d) TiSn2300, e) TiSn2500 and f) TiSn2600. Inset are hysteresis loops. Figure S3. Pore area distribution of a) TiSn401, b) TiSn403, c) TiSn405, d) TiSn410, e) TiSn420 and f) TiSn430. Inset are hysteresis loops. Figure S4. Selected Area Electron Diffraction (SAED) of sample a) TiSn2050 - anatase, b) TiSn2100 - anatase, c) TiSn2200 - anatase, d) TiSn2300 - brookite, e) TiSn2400 - anatase and f) TiSn2600 - rutile. Figure S5. Selected Area Electron Diffraction (SAED) of sample a) TiSn401 - anatase, b) TiSn405 - anatase, c) TiSn407 - anatase and brookite, d) TiSn410 - rutile, e) TiSn420 - rutile and f) TiSn430 - rutile. Figure S6. UV-VIS spectra of series samples a) Sn2+ doped TiO2 and b) Sn4+ doped TiO2. Figure S7. Band-gap energy of titanium oxides prepared in the presence of a) Sn2+ and b) Sn4+. Table S5. Rate constant k k, k1 and k2 of tin doped titania. [file 1752-153X-6-113-S1.pdf]

Supporting Informations

Hydrogen peroxide route to Sn-doped titania catalysts activated by visible light

[1,2] Václav Štengl*, [1,2] Tomáš Matys Grygar, [1,2] Jiří Henych, [3] Martin Kormunda

[1] Department of Solid State Chemistry and Analytical Laboratory

stengl@iic.cas.cz, phone: +420 / 266 173 193, fax: +420 / 220 94 15 02

Institute of Inorganic Chemistry AS CR, v.v.i. 250 68 Řež, Czech Republic

[2]Faculty of the Environment, University of Jan Evangelista Purkyně, Králova Výšina 7, 400 96 Ústí nad Labem, Czech Republic

[3] Department of Physics, Faculty of Science, J. E. Purkyně University, České mládeže 8, 400 96 Ústí nad Labem., Czech Republic

Table S1. Cell parameters *a*, *b* and *c* of anatase, rutile and brookite doped with $SnCl_2$

Sample	Anatase a [nm]	Anatase c [nm]	Rutile a [nm]	Rutile c [nm]	Brookite a [nm]	Brookite b [nm]	Brookite c [nm]
TiSn2025	3.7963	9.5220	-	-	-	-	-
TiSn2050	3.7948	9.5119	-	-	9.0814	5.4968	5.1677
TiSn2100	3.7958	9.5140	-	-	9.0393	5.5036	5.1611
TiSn2200	3.7981	9.5210	4.6425	2.9913	-	-	-
TiSn2300	3.8027	9.5333	4.6429	2.9927	9.0839	5.5015	5.1981
TiSn2400	3.8024	9.5370	4.6447	2.9945	-	-	-
TiSn2500	-	-	4.6473	2.9947	-	-	-
TiSn2600	-	-	4.6493	2.9947	-	-	-

Table S2. Cell parameters a, b and c of anatase, rutile and brookite doped with $SnCl_4$

Sample	Anatase a [nm]	Anatase c [nm]	Rutile a [nm]	Rutile c [nm]	Brookite a [nm]	Brookite b [nm]	Brookite c [nm]
TiSn401	3.7937	9.5120	4.6111	2.9586	9.0661	5.4298	5.1687
TiSn403	3.7933	9.5195	4.6110	2.9686	9.0614	5.4360	5.1771
TiSn405	3.7937	9.5173	4.6137	2.9754	9.0596	5.4481	5.1861
TiSn407	3.7906	9.5221	4.6135	2.9738	9.0557	5.4515	5.1711
TiSn410	-	-	4.6123	2.9735	9.0558	5.4637	5.1663

TiSn420	-	-	4.6129	2.9770	-	-	-
TiSn430	-	-	4.6112	2.9882	-	-	-

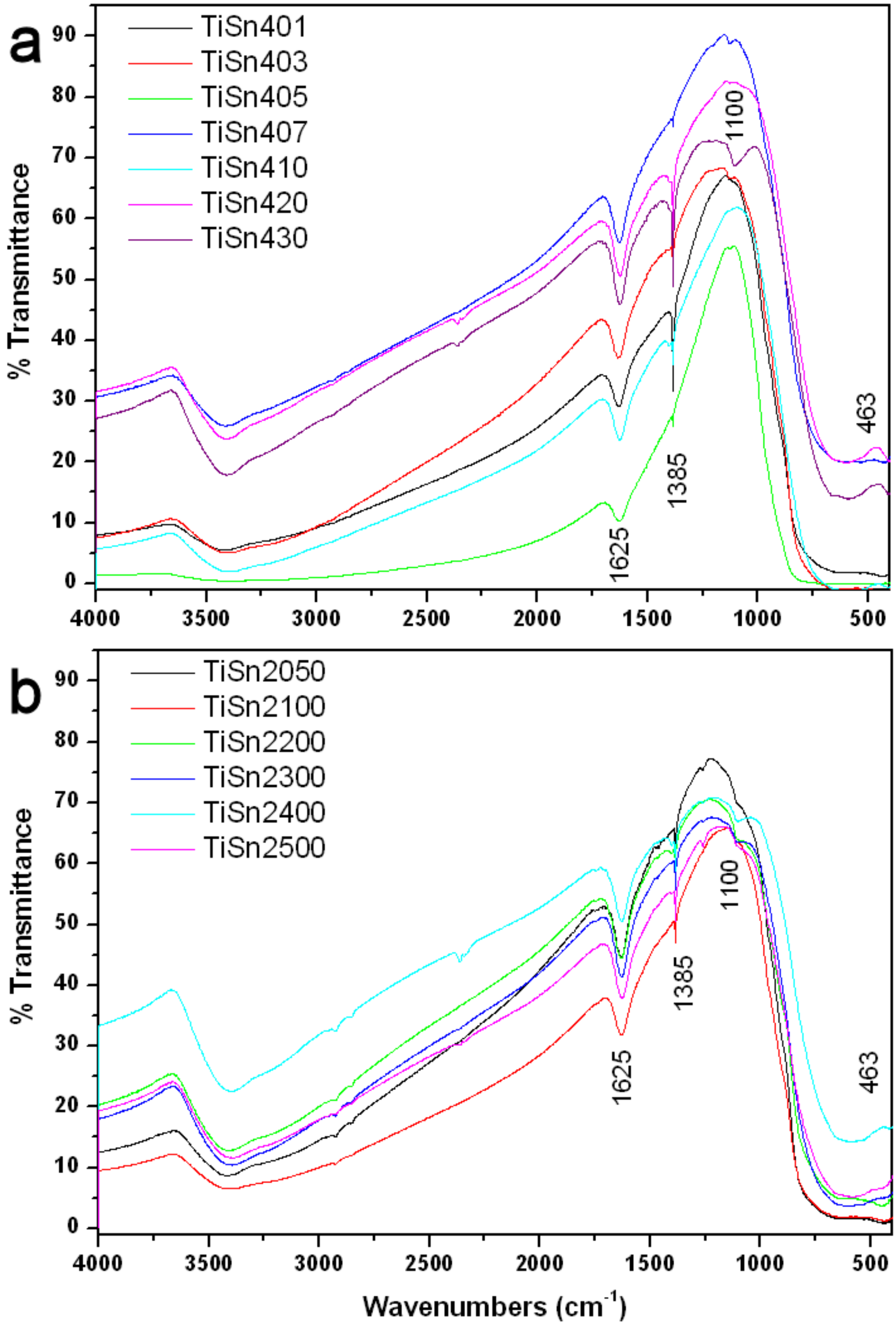

Figure S1. Infrared spectra of series samples a) Sn^{4+} doped TiO_2 and b) Sn^{2+} doped TiO_2

Table S3. Atomic concentrations of elements from XPS measurements

Sample	O [at.%]	Sn [at.%]	C [at.%]	Ti [at.%]	O/Ti
TiSn403	66.30	0.87	5.19	27.64	2.40
TiSn410	67.69	4.65	2.34	25.32	2.67
TiSn2100	69.47	0.85	1.91	27.78	2.50
TiSn2300	68.47	4.46	1.88	25.19	2.72

Table S4. Binding energies and FWHM of Sn 3d and Ti 2p peaks

	Sn 3d				Ti 2p				Ti satellites	
	3/2		5/2		1/2		3/2			
Sample	B.E. [eV]	FWHM [eV]	B.E. [eV]	FWHM [eV]	B.E. [eV]	FWHM [eV]	B.E. [eV]	FWHM [eV]	B.E. [eV]	B.E. [eV]
TiSn403	495.4	1.7	486.9	1.9	464.7	2.5	459.0	1.6	472.4	477.8
TiSn410	495.3	1.8	486.9	2.0	464.6	2.5	459.0	1.6	472.5	477.2
TiSn2100	495.4	1.7	487.0	2.3	464.7	2.4	459.0	1.5	472.4	477.8
TiSn2300	495.4	1.8	487.0	1.9	464.7	2.5	459.0	1.6	472.4	477.2

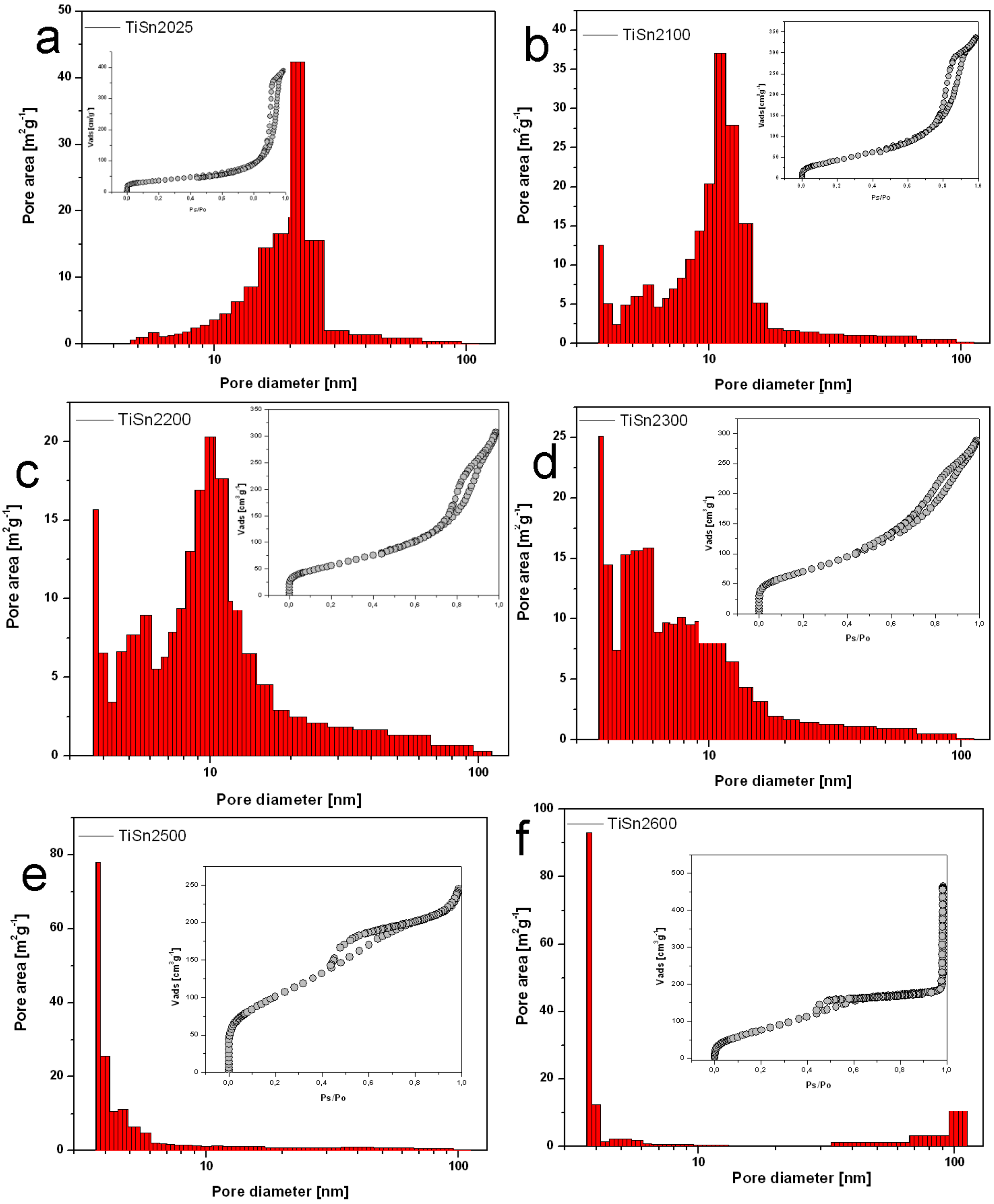

Figure S2. Pore area distribution of a) TiSn2025, b) TiSn2100, c) TiSn2200, d) TiSn2300, e) TiSn2500 and f) TiSn2600. Inset are hysteresis loops

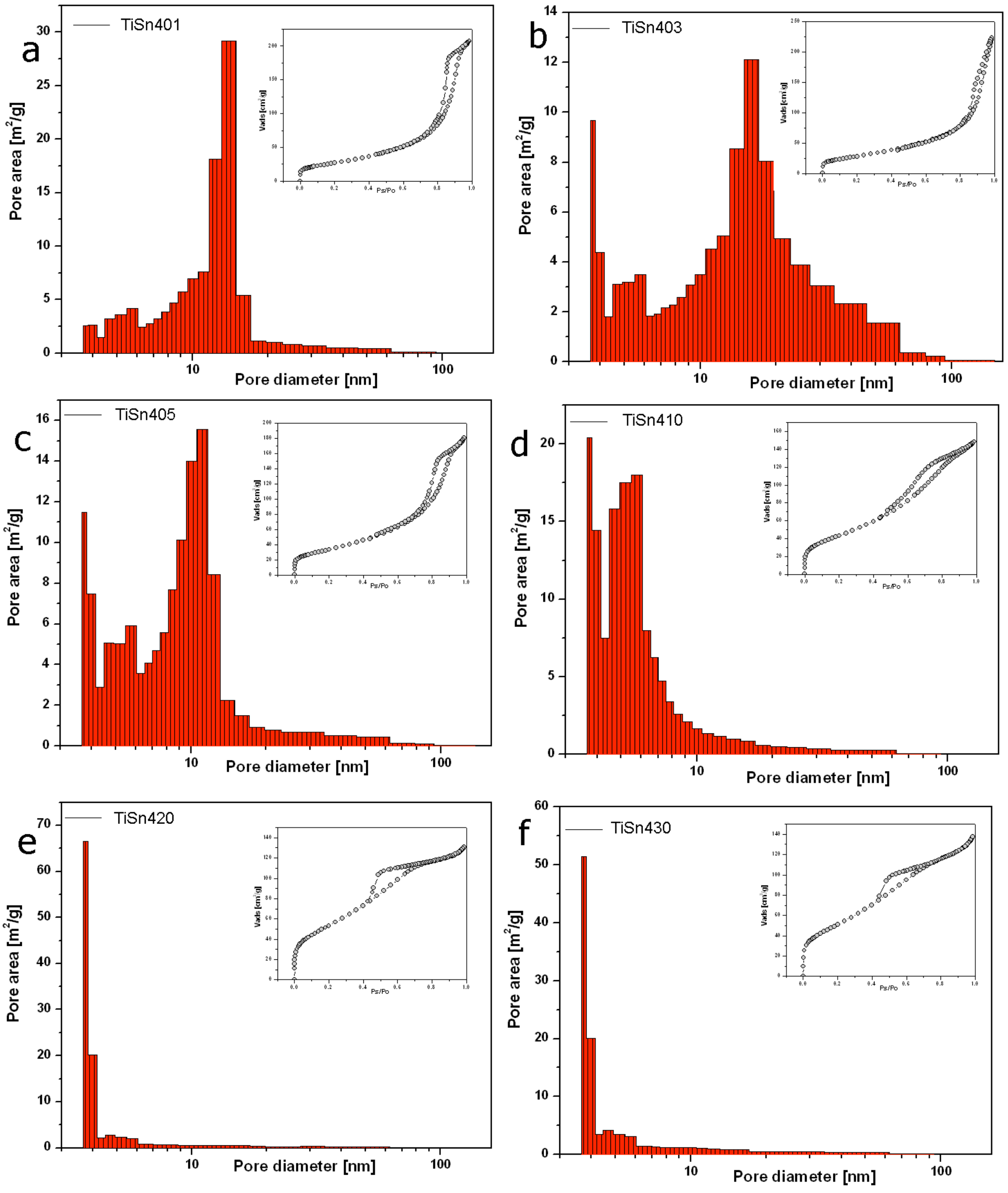

Figure S3. Pore area distribution of a) TiSn401, b) TiSn403, c) TiSn405, d) TiSn410, e) TiSn420 and f) TiSn430. Inset are hysteresis loops

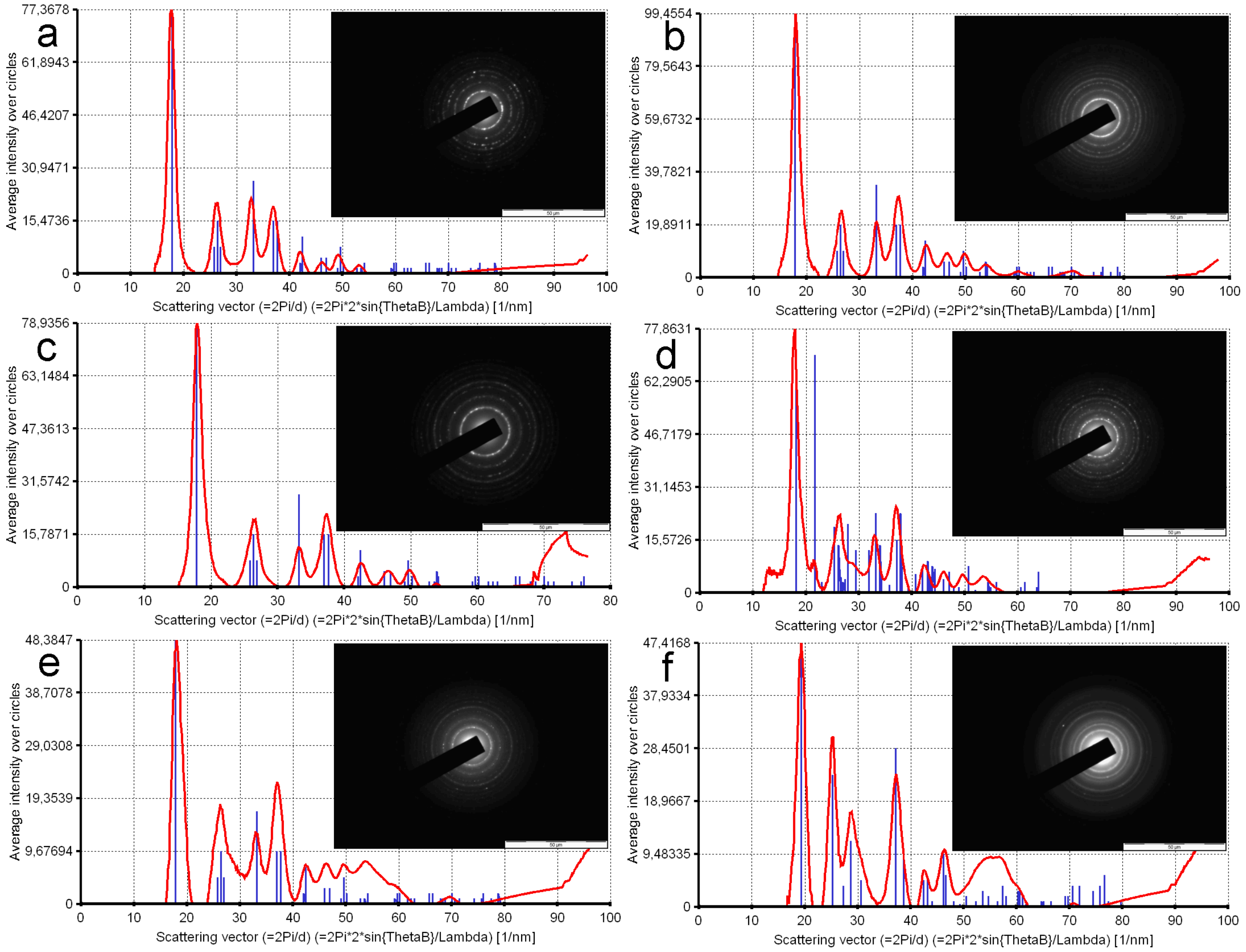

Figure S4. Selected Area Electron Diffraction (SAED) of sample a) TiSn2050 - anatase, b) TiSn2100 - anatase, c) TiSn2200 - anatase, d) TiSn2300 - brookite, e) TiSn2400 - anatase and f) TiSn2600 - rutile

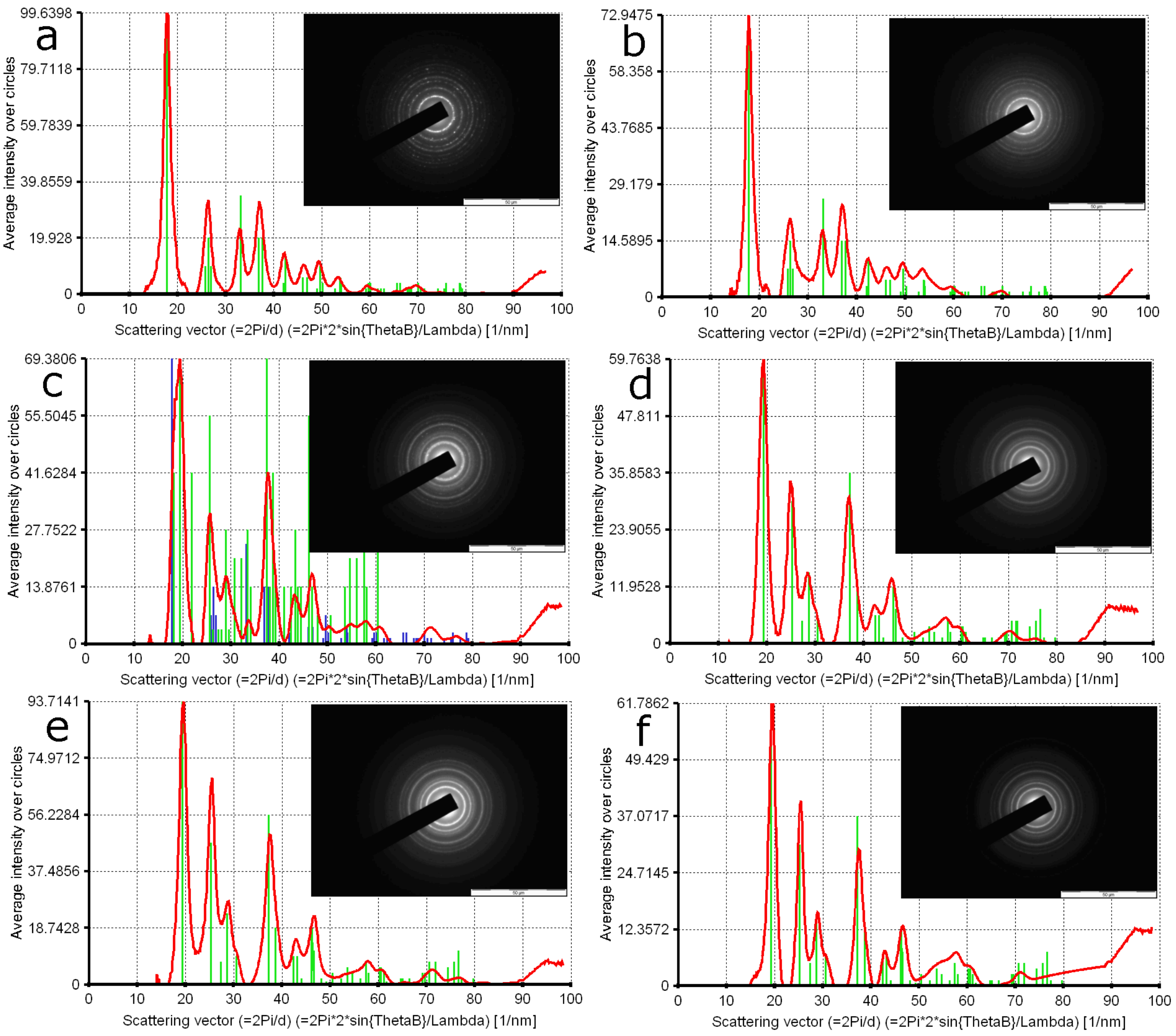

Figure S5. Selected Area Electron Diffraction (SAED) of sample a) TiSn401 - anatase, b) TiSn405 - anatase, c) TiSn407 - anatase and brookite, d) TiSn410 - rutile, e) TiSn420 - rutile and f) TiSn430 - rutile

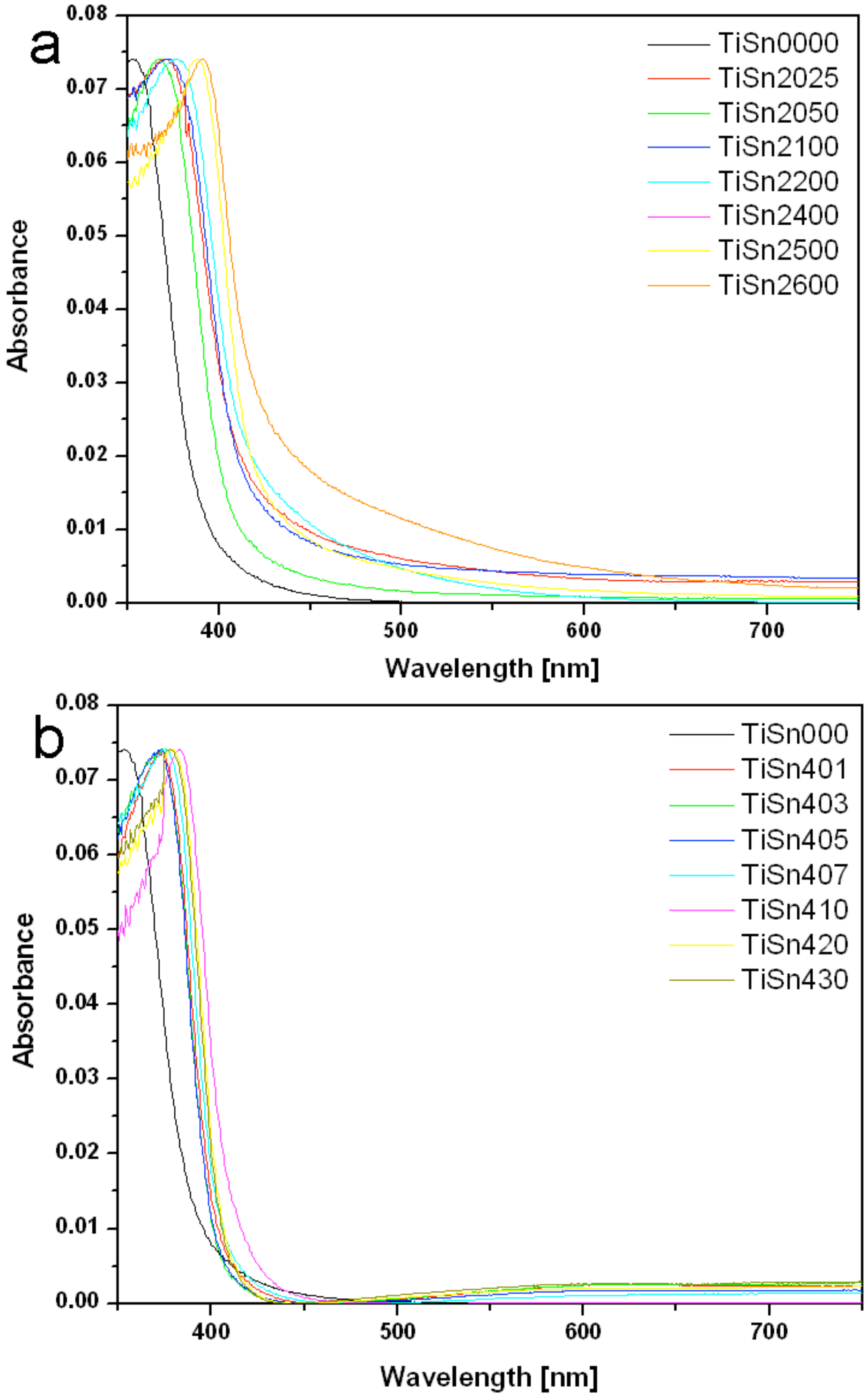

Figure S6. UV-VIS spectra of series samples a) Sn^{2+} doped TiO_2 and b) Sn^{4+} doped TiO_2

The minimum wavelength required to promote an electron depends upon the band-gap energy E_{bg} which is commonly estimated from UV-Vis absorption spectra by the linear extrapolation of the absorption coefficient to zero using the following equation:

$$\alpha(h\nu) = A(h\nu-E_{bg})^n \qquad (1)$$

where A is the absorption according to eq. (1), B is absorption coefficient, hν is the photon energy in eV calculated from the wavelength λ in nm [1], [2].

$$h\nu=1239/\lambda \qquad (2)$$

In the case of n=2 the fundamental absorption of photocatalyst crystals is due to a direct transition between bands, while for the indirect transition between bands has the n value of ½ [3],[4]. The energy of the band gap is calculated by extrapolating a straight line to the abscissa axis, when α is zero, then E_{bg} = hν [5].

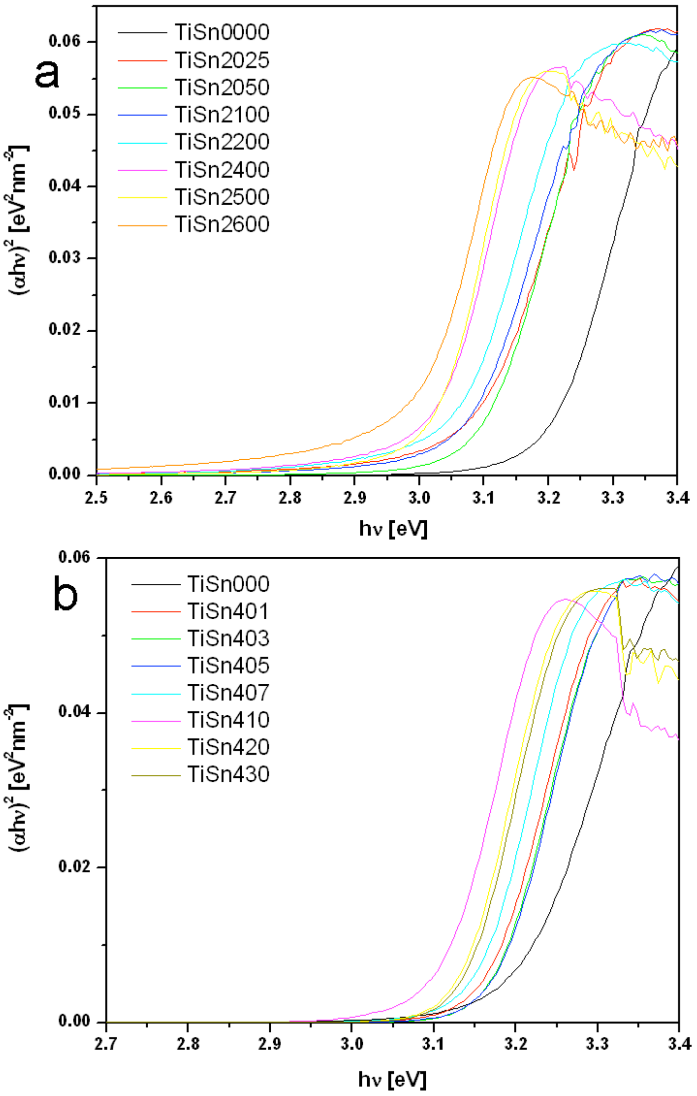

Figure S7. Band-gap energy of titanium oxides prepared in the presence of a) Sn^{2+} and b) Sn^{4+}

**Table S5. Rate constant k, k_1 and k_2 of tin doped titania.**						
**$SnCl_4$**				**$SnCl_2 \cdot 2H_2O$**		
**Samples**	**k OII 365 nm [min^{-1}]**	**k_1 OII 400 nm [min^{-1}]**	**k_2 OII 400 nm [min^{-1}]**	**Samples**	**k OII 365 nm [min^{-1}]**	**k OII 400 nm [min^{-1}]**
TiSn401	0.02119	0.02843	0.00295	TiSn2025	0.0520	0.00374
TiSn403	0.03286	0.04401	0.00321	TiSn2050	0.0837	0.00767
TiSn405	0.03721	0.04253	0.00404	TiSn2100	0.0659	0.01078
TiSn407	0.04123	0.04404	0.00664	TiSn2200	0.0549	0.00671
TiSn410	**0.09418**	**0.08342**	**0.00926**	TiSn2300	**0.1259**	0.01717
TiSn420	0.08144	0.04742	0.00505	TiSn2400	0.1109	0.01409
TiSn430	0.05415	0.05904	0.00298	TiSn2500	0.0834	**0.03595**
TiSn000	0.00730	0.00200	**-**	TiSn2600	0.0666	0.01586

References:

[1] H. Yuan, J. Xu, International Journal of Chemical Engineering and Applications 1 (2010) 241- 246.

[2] K.M. Reddy, S.V. Manorama, A.R. Reddy, Materials Chemistry and Physics 78 (2003) 239-245.

[3] D. Reyes-Coronado, G. Rodriguez-Gattorno, M.E. Espinosa-Pesqueira, C. Cab, R. de Coss, G. Oskam, Nanotechnology 19 (2008).

[4] N. Serpone, D. Lawless, R. Khairutdinov, Journal of Physical Chemistry 99 (1995) 16646-16654.

[5] E. Sanchez, T. Lopez, Materials Letters 25 (1995) 271-275.
